# The genome sequence of the China Limpet, *Patella ulyssiponensis* Gmelin, 1791 (Patellidae)

**DOI:** 10.12688/wellcomeopenres.24681.1

**Published:** 2025-08-11

**Authors:** Nova Mieszkowska, Stephen J Hawkins, Helena B S M Côrte-Real, Louise B Firth, Marine Biological Association Genome Acquisition Lab, Darwin Tree of Life Barcoding collective, Wellcome Sanger Institute Tree of Life Management, Samples and Laboratory team, Wellcome Sanger Institute Scientific Operations: Sequencing Operations, Wellcome Sanger Institute Tree of Life Core Informatics team, Tree of Life Core Informatics collective, Darwin Tree of Life Consortium

**Affiliations:** 1 The Marine Biological Association, Plymouth, England, UK; 2 University of Liverpool, Liverpool, England, UK; 3 University of Southampton, Southampton, England, UK; 4 University of Cork, Cork, Ireland; 5 University of Plymouth, Plymouth, England, UK

**Keywords:** Patella ulyssiponensis, China Limpet, genome sequence, chromosomal, Mollusca

## Abstract

We present a genome assembly from a specimen of *Patella ulyssiponensis* (China Limpet; Mollusca; Gastropoda; Patellidae). The genome sequence has a total length of 693.56 megabases. Most of the assembly (99.78%) is scaffolded into 8 chromosomal pseudomolecules. The mitochondrial genome has also been assembled, with a length of 14.94 kilobases. Gene annotation of this assembly on Ensembl identified 21 151 protein-coding genes.

## Species taxonomy

Eukaryota; Opisthokonta; Metazoa; Eumetazoa; Bilateria; Protostomia; Spiralia; Lophotrochozoa; Mollusca; Gastropoda; Patellogastropoda; Patelloidea; Patellidae; *Patella*; *Patella ulyssiponensis* Gmelin, 1791 (NCBI:txid334708)

## Background

*Patella ulyssiponensis* Gmelin, 1791 (often referred to as *P. aspera* Röding, 1798) occurs on exposed rocky shores from Mauritania to Norway, including all suitable shores of Ireland and the British Isles (except the soft coasts between Kent and Flamborough Head) and in the Black Sea and Mediterranean. Its wide range led to extensive synonymy and shifting names based on shell characters ( Christiaens, 1973 listed 53). Early names ( *P. ulyssiponensis* Gmelin, 1791; *P. aspera* Röding, 1798; *P. athletica* Bean, 1844) were distinguished or merged by 19th- and early 20th-century authors ( Dautzenberg, 1906; Jeffreys, 1865; Powell, 1973). Genetic methods – from allozyme electrophoresis ( Gaffney, 1980) to DNA sequencing ( Koufopanou *et al.*, 1999; Sá-Pinto *et al.*, 2005; Sá-Pinto *et al.*, 2012; Weber & Hawkins, 2005) – have confirmed that *P. ulyssiponensis* (European mainland) is distinct from *P. aspera* (Azores, Canaries, Madeira). The status of Black Sea and southern Atlantic (St Helena, Angola) populations remains uncertain.

*Patella ulyssiponensis* occupies low-shore open rock and mid-shore pools, often where crustose calcareous algae (CCA) grow ( Delany *et al.*, 1998; Firth & Crowe, 2008). On very exposed shores it reappears under the *Laminaria digitata* canopy, where wave action limits turf algae ( Hawkins & Harkin, 1985). Limpet grazing maintains bare patches on CCA, preventing turfs and possibly aiding CCA growth; larvae may preferentially settle on CCA ( Bowman, 1981; Firth *et al.*, 2023).

This species is a protandrous hermaphrodite, maturing first as male and often later changing to female ( Thompson, 1980). In related species sex change can depend on density; this has yet to be tested in *P. ulyssiponensis*. In northern Portugal there are two spawning peaks (September to January and March to June, Ribeiro *et al.*, 2009), whereas in Ireland and the British Isles there is a single annual peak, with gamete maturation in early summer and spawning in October ( Thompson, 1979).

Transplant experiments show that *P. ulyssiponensis* can shift red algal turf limits upward on the shore ( Boaventura *et al.*, 2002). In Portugal it co-exists in pools with juvenile *P. depressa* and the pulmonate *Siphonaria pectinata*, which later move onto open rock; by contrast, *P. ulyssiponensis* remains in pools, controlling macroalgae and keeping CCA free of epibionts ( Firth *et al.*, 2023; O’Connor & Crowe, 2005).

Beyond grazing, this limpet bioerodes softer rock, creating microhabitats that support diverse species ( Cooper *et al.*, 2025). Its empty shells also shelter macroalgae from other grazers. Together, these activities make *P. ulyssiponensis* an ecosystem engineer with both habitat-forming and habitat-modifying roles.

**Figure 1.**
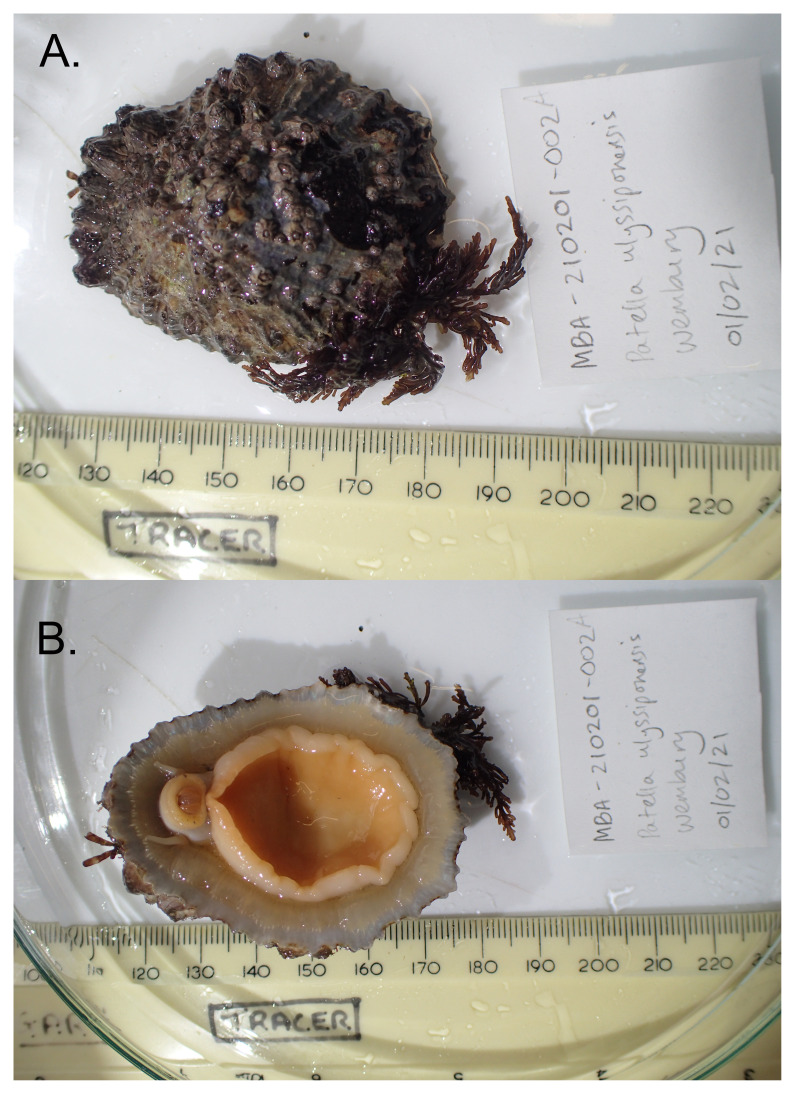
Photograph of the *Patella ulyssiponensis* (xgPatUlys2) specimen from which samples were taken for genome sequencing.

The genome of *Patella ulyssiponensis* presented here was assembled using the Tree of Life pipeline from a specimen collected in Church Reef, Wembury, Devon, United Kingdom ( Figure 1).

## Methods

### Sample acquisition and DNA barcoding

The specimen used for genome sequencing was an adult *Patella ulyssiponensis* (specimen ID MBA-210201-002A, ToLID xgPatUlys2; Figure 1), collected from Church Reef, Wembury, Devon, United Kingdom (latitude 50.3155, longitude –4.0829) on 2021-02-01. The specimen was collected and identified by Nova Mieszkowska (Marine Biological Association). A second specimen was used for Hi-C and RNA sequencing (specimen ID MBA-200706-001A, ToLID xgPatUlys1). It was collected from Godrevy, Cornwall, United Kingdom (latitude 50.2379, longitude –5.3961) on 2020-07-06. The specimen was collected and identified by Nova Mieszkowska and Rob Mrowicki.

The initial identification was verified by an additional DNA barcoding process according to the framework developed by Twyford *et al.* (2024). A small sample was dissected from the specimen and stored in ethanol, while the remaining parts were shipped on dry ice to the Wellcome Sanger Institute (WSI). The tissue was lysed, the COI marker region was amplified by PCR, and amplicons were sequenced and compared to the BOLD database, confirming the species identification ( Crowley *et al.*, 2023). Following whole genome sequence generation, the relevant DNA barcode region was also used alongside the initial barcoding data for sample tracking at the WSI ( Twyford *et al.*, 2024). The standard operating procedures for Darwin Tree of Life barcoding are available on protocols.io.

### Nucleic acid extraction

Protocols for high molecular weight (HMW) DNA extraction developed at the Wellcome Sanger Institute (WSI) Tree of Life Core Laboratory are available on protocols.io ( Howard *et al.*, 2025). The xgPatUlys2 sample was weighed and triaged to determine the appropriate extraction protocol. Tissue from the muscle was homogenised by cryogenic disruption using the Covaris cryoPREP ^®^ Automated Dry Pulverizer. HMW DNA was extracted using the Automated MagAttract v2 protocol. DNA was sheared into an average fragment size of 12–20 kb following the Megaruptor®3 for LI PacBio protocol. Sheared DNA was purified by automated SPRI (solid-phase reversible immobilisation). The concentration of the sheared and purified DNA was assessed using a Nanodrop spectrophotometer and Qubit Fluorometer using the Qubit dsDNA High Sensitivity Assay kit. Fragment size distribution was evaluated by running the sample on the FemtoPulse system.

RNA was extracted from muscle tissue of xgPatUlys1 in the Tree of Life Laboratory at the WSI using the RNA Extraction: Automated MagMax™ *mir*Vana protocol. The RNA concentration was assessed using a Nanodrop spectrophotometer and a Qubit Fluorometer using the Qubit RNA Broad-Range Assay kit. Analysis of the integrity of the RNA was done using the Agilent RNA 6000 Pico Kit and Eukaryotic Total RNA assay.

### PacBio HiFi library preparation and sequencing

Library preparation and sequencing were performed at the WSI Scientific Operations core. Libraries were prepared using the SMRTbell Prep Kit 3.0 (Pacific Biosciences, California, USA), following the manufacturer’s instructions. The kit includes the reagents required for end repair/A-tailing, adapter ligation, post-ligation SMRTbell bead cleanup, and nuclease treatment. Size-selection and clean-up were carried out using diluted AMPure PB beads (Pacific Biosciences). DNA concentration was quantified using the Qubit Fluorometer v4.0 (ThermoFisher Scientific) with Qubit 1X dsDNA HS assay kit and the final library fragment size analysis was carried out using the Agilent Femto Pulse Automated Pulsed Field CE Instrument (Agilent Technologies) and the gDNA 55kb BAC analysis kit.

The sample was sequenced using the Sequel IIe system. The concentration of the library loaded onto the Sequel IIe was in the range 40–135 pM. The SMRT link software, a PacBio web-based end-to-end workflow manager, was used to set-up and monitor the run, and to perform primary and secondary analysis of the data upon completion.

### Hi-C

***Sample preparation and crosslinking***

The Hi-C sample was prepared from 20–50 mg of frozen muscle tissue of the xgPatUlys1 sample using the Arima-HiC v2 kit (Arima Genomics). Following the manufacturer’s instructions, tissue was fixed and DNA crosslinked using TC buffer to a final formaldehyde concentration of 2%. The tissue was homogenised using the Diagnocine Power Masher-II. Crosslinked DNA was digested with a restriction enzyme master mix, biotinylated, and ligated. Clean-up was performed with SPRISelect beads before library preparation. DNA concentration was measured with the Qubit Fluorometer (Thermo Fisher Scientific) and Qubit HS Assay Kit. The biotinylation percentage was estimated using the Arima-HiC v2 QC beads.

***Hi-C library preparation and sequencing***

Biotinylated DNA constructs were fragmented using a Covaris E220 sonicator and size selected to 400–600 bp using SPRISelect beads. DNA was enriched with Arima-HiC v2 kit Enrichment beads. End repair, A-tailing, and adapter ligation were carried out with the NEBNext Ultra II DNA Library Prep Kit (New England Biolabs), following a modified protocol where library preparation occurs while DNA remains bound to the Enrichment beads. Library amplification was performed using KAPA HiFi HotStart mix and a custom Unique Dual Index (UDI) barcode set (Integrated DNA Technologies). Depending on sample concentration and biotinylation percentage determined at the crosslinking stage, libraries were amplified with 10–16 PCR cycles. Post-PCR clean-up was performed with SPRISelect beads. Libraries were quantified using the AccuClear Ultra High Sensitivity dsDNA Standards Assay Kit (Biotium) and a FLUOstar Omega plate reader (BMG Labtech).

Prior to sequencing, libraries were normalised to 10 ng/μL. Normalised libraries were quantified again and equimolar and/or weighted 2.8 nM pools. Pool concentrations were checked using the Agilent 4200 TapeStation (Agilent) with High Sensitivity D500 reagents before sequencing. Sequencing was performed using paired-end 150 bp reads on the Illumina NovaSeq 6000.

### RNA library preparation and sequencing

Libraries were prepared using the NEBNext ^®^ Ultra™ II Directional RNA Library Prep Kit for Illumina (New England Biolabs), following the manufacturer’s instructions. Poly(A) mRNA in the total RNA solution was isolated using oligo(dT) beads, converted to cDNA, and uniquely indexed; 14 PCR cycles were performed. Libraries were size-selected to produce fragment sizes of 100–300 bp. Libraries were quantified, normalised, pooled to a final concentration of 2.8 nM, and diluted to 150 pM for loading. Sequencing was carried out on the Illumina NovaSeq 6000 to generate 150-bp paired-end reads.

### Genome assembly

Prior to assembly of the PacBio HiFi reads, a database of *k*-mer counts ( *k* = 31) was generated from the filtered reads using FastK. GenomeScope2 ( Ranallo-Benavidez *et al.*, 2020) was used to analyse the *k*-mer frequency distributions, providing estimates of genome size, heterozygosity, and repeat content.

The HiFi reads were assembled using Hifiasm ( Cheng *et al.*, 2021) with the --primary option. Haplotypic duplications were identified and removed using purge_dups ( Guan *et al.*, 2020). The Hi-C reads ( Rao *et al.*, 2014) were mapped to the primary contigs using bwa-mem2 ( Vasimuddin *et al.*, 2019), and the contigs were scaffolded in YaHS ( Zhou *et al.*, 2023) with the --break option for handling potential misassemblies. The scaffolded assemblies were evaluated using Gfastats ( Formenti *et al.*, 2022), BUSCO ( Manni *et al.*, 2021) and MERQURY.FK ( Rhie *et al.*, 2020).

The mitochondrial genome was assembled using MitoHiFi ( Uliano-Silva *et al.*, 2023), which runs MitoFinder ( Allio *et al.*, 2020) and uses these annotations to select the final mitochondrial contig and to ensure the general quality of the sequence.

### Assembly curation

The assembly was decontaminated using the Assembly Screen for Cobionts and Contaminants ( ASCC) pipeline. TreeVal was used to generate the flat files and maps for use in curation. Manual curation was conducted primarily in PretextView and HiGlass ( Kerpedjiev *et al.*, 2018). Scaffolds were visually inspected and corrected as described by Howe *et al*. (2021). Manual corrections included 32 breaks, 29 joins, and removal of 12 haplotypic duplications. The curation process is documented at https://gitlab.com/wtsi-grit/rapid-curation. PretextSnapshot was used to generate a Hi-C contact map of the final assembly.

### Assembly quality assessment

The Merqury.FK tool ( Rhie *et al.*, 2020), run in a Singularity container ( Kurtzer *et al.*, 2017), was used to evaluate *k*-mer completeness and assembly quality for the primary and alternate haplotypes using the *k*-mer databases ( *k* = 31) computed prior to genome assembly. The analysis outputs included assembly QV scores and completeness statistics.

The genome was analysed using the BlobToolKit pipeline, a Nextflow implementation of the earlier Snakemake BlobToolKit pipeline ( Challis *et al.*, 2020). The pipeline aligns PacBio reads using minimap2 ( Li, 2018) and SAMtools ( Danecek *et al.*, 2021) to generate coverage tracks. Simultaneously, it queries the GoaT database ( Challis *et al.*, 2023) to identify relevant BUSCO lineages and runs BUSCO ( Manni *et al.*, 2021). For the three domain-level BUSCO lineages, BUSCO genes are aligned to the UniProt Reference Proteomes database ( Bateman *et al.*, 2023) using DIAMOND blastp ( Buchfink *et al.*, 2021). The genome is divided into chunks based on the density of BUSCO genes from the closest taxonomic lineage, and each chunk is aligned to the UniProt Reference Proteomes database with DIAMOND blastx. Sequences without hits are chunked using seqtk and aligned to the NT database with blastn ( Altschul *et al.*, 1990). The BlobToolKit suite consolidates all outputs into a blobdir for visualisation. The BlobToolKit pipeline was developed using nf-core tooling ( Ewels *et al.*, 2020) and MultiQC ( Ewels *et al.*, 2016), with package management via Conda and Bioconda ( Grüning *et al.*, 2018), and containerisation through Docker ( Merkel, 2014) and Singularity ( Kurtzer *et al.*, 2017).

## Genome sequence report

### Sequence data

**Figure 2.**
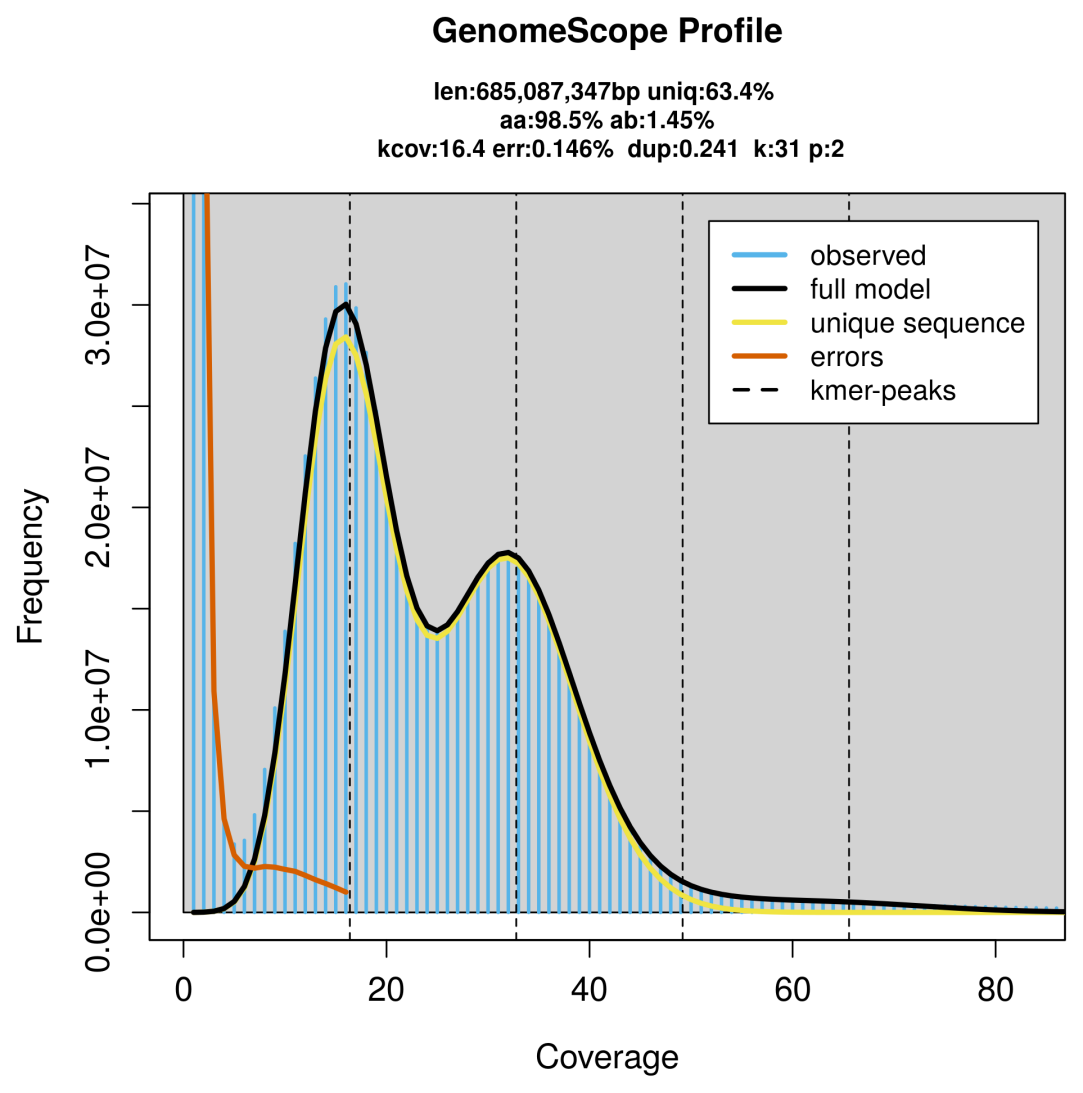
Frequency distribution of *k*-mers generated using GenomeScope2. The plot shows observed and modelled *k*-mer spectra, providing estimates of genome size, heterozygosity, and repeat content based on unassembled sequencing reads.

The genome of a specimen of *Patella ulyssiponensis* was sequenced using Pacific Biosciences single-molecule HiFi long reads, generating 23.58 Gb (gigabases) from 2.68 million reads, which were used to assemble the genome. GenomeScope2.0 analysis estimated the haploid genome size at 685.09 Mb, with a heterozygosity of 1.45% and repeat content of 36.76% ( Figure 2). These estimates guided expectations for the assembly. Based on the estimated genome size, the sequencing data provided approximately 33× coverage. Hi-C sequencing produced 96.42 Gb from 638.57 million reads, which were used to scaffold the assembly. RNA sequencing data were also generated and are available in public sequence repositories. Table 1 summarises the specimen and sequencing details.

**Table 1.** Specimen and sequencing data for BioProject PRJEB63446.

Platform	PacBio HiFi	Hi-C	RNA-seq
**ToLID**	xgPatUlys2	xgPatUlys1	xgPatUlys1
**Specimen ID**	MBA-210201-002A	MBA-200706-001A	MBA-200706-001A
**BioSample (source ** **individual)**	SAMEA8419699	SAMEA7536242	SAMEA7536242
**BioSample (tissue)**	SAMEA8419982	SAMEA7536328	SAMEA7536329
**Tissue**	muscle	muscle	muscle
**Instrument**	Sequel IIe	Illumina NovaSeq 6000	Illumina NovaSeq 6000
**Run accessions**	ERR11593810; ERR11593809	ERR11606324	ERR11606323
**Read count total**	2.68 million	638.57 million	44.63 million
**Base count total**	23.58 Gb	96.42 Gb	6.74 Gb

### Assembly statistics

The primary haplotype was assembled, and contigs corresponding to an alternate haplotype were also deposited in INSDC databases. The final assembly has a total length of 693.56 Mb in 27 scaffolds, with 73 gaps, and a scaffold N50 of 86.69 Mb ( Table 2).

**Table 2.** Genome assembly statistics.

Genome assembly	Primary assembly
**Assembly name**	xgPatUlys2.1
**Assembly accession**	GCA_963678685.1
**Alternate haplotype accession**	GCA_963678665.1
**Assembly level**	chromosome
**Span (Mb)**	693.56
**Number of chromosomes**	8
**Number of contigs**	100
**Contig N50**	16.75 Mb
**Number of scaffolds**	27
**Scaffold N50**	86.69 Mb
**Longest scaffold length (Mb)**	104.01
**Organelles**	Mitochondrial genome: 14.94 kb

**Figure 3.**
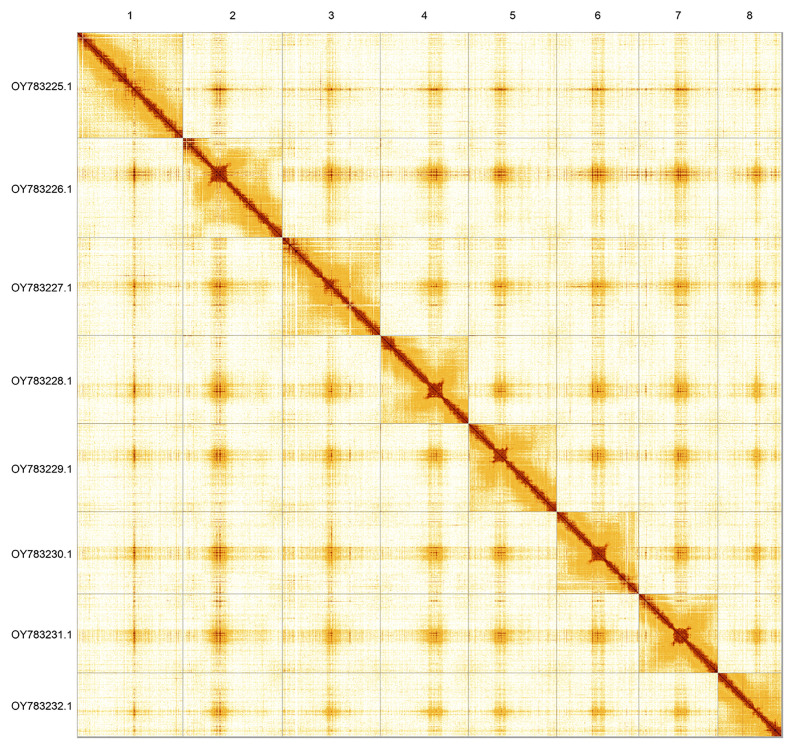
Hi-C contact map of the *Patella ulyssiponensis* genome assembly. Assembled chromosomes are shown in order of size and labelled along the axes. The plot was generated using PretextSnapshot.

Most of the assembly sequence (99.78%) was assigned to 8 chromosomal-level scaffolds. These chromosome-level scaffolds, confirmed by Hi-C data, are named according to size ( Figure 3; Table 3).

**Table 3.** Chromosomal pseudomolecules in the primary genome assembly of *Patella ulyssiponensis* xgPatUlys2.

INSDC accession	Molecule	Length (Mb)	GC%
OY783225.1	1	104.01	36.50
OY783226.1	2	97.66	36
OY783227.1	3	96.22	36.50
OY783228.1	4	86.69	36
OY783229.1	5	86.65	36
OY783230.1	6	80.63	36.50
OY783231.1	7	77.60	36.50
OY783232.1	8	62.57	36.50

The mitochondrial genome was also assembled. This sequence is included as a contig in the multifasta file of the genome submission and as a standalone record.

### Assembly quality metrics

**Figure 4.**
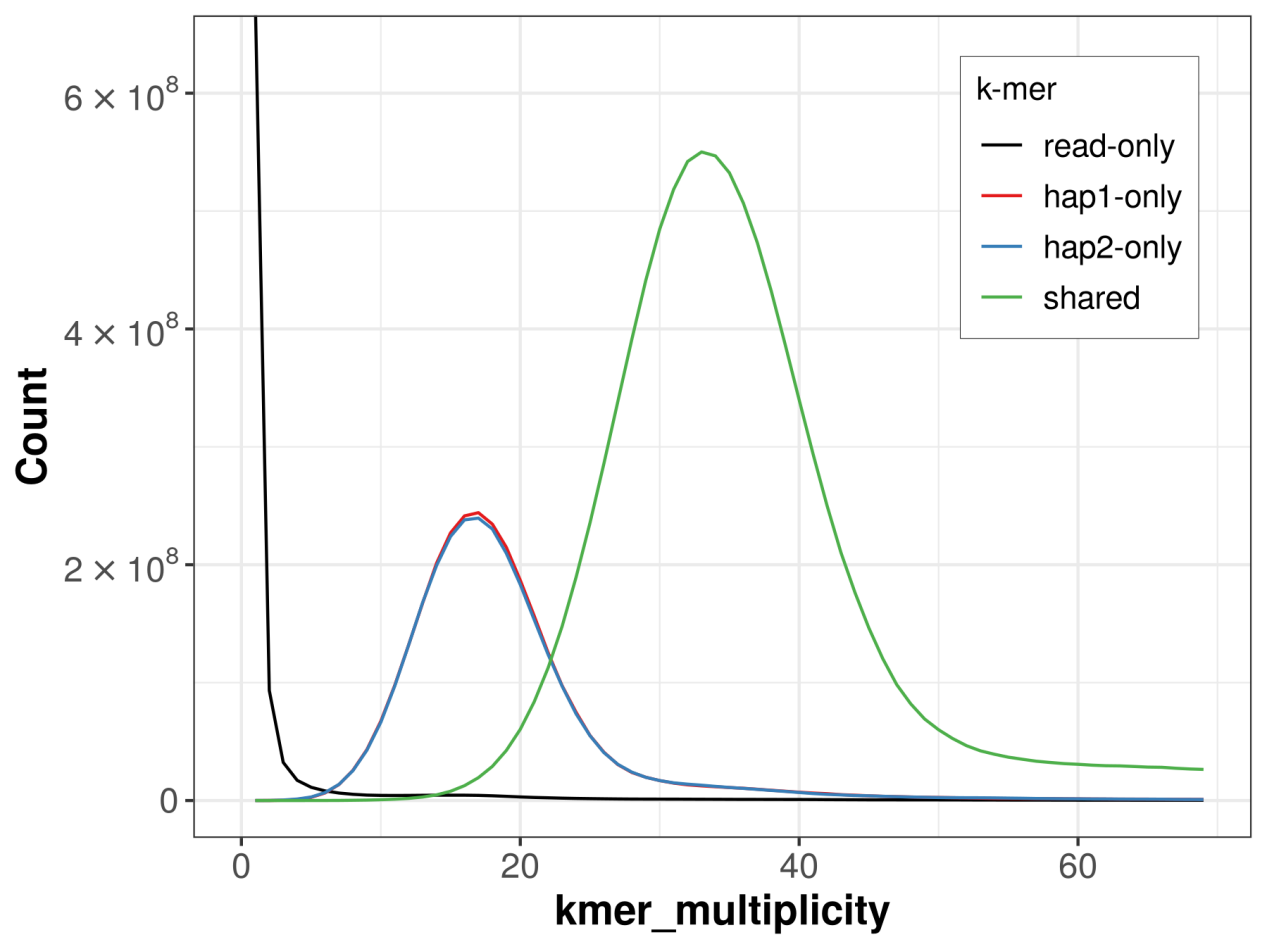
Evaluation of *k*-mer completeness using MerquryFK. This plot illustrates the recovery of *k*-mers from the original read data in the final assemblies. The horizontal axis represents *k*-mer multiplicity, and the vertical axis shows the number of *k*-mers. The black curve represents *k*-mers that appear in the reads but are not assembled. The green curve (the homozygous peak) corresponds to *k*-mers shared by both haplotypes and the red and blue curves (the heterozygous peaks) show *k*-mers found only in one of the haplotypes.

**Figure 5.**
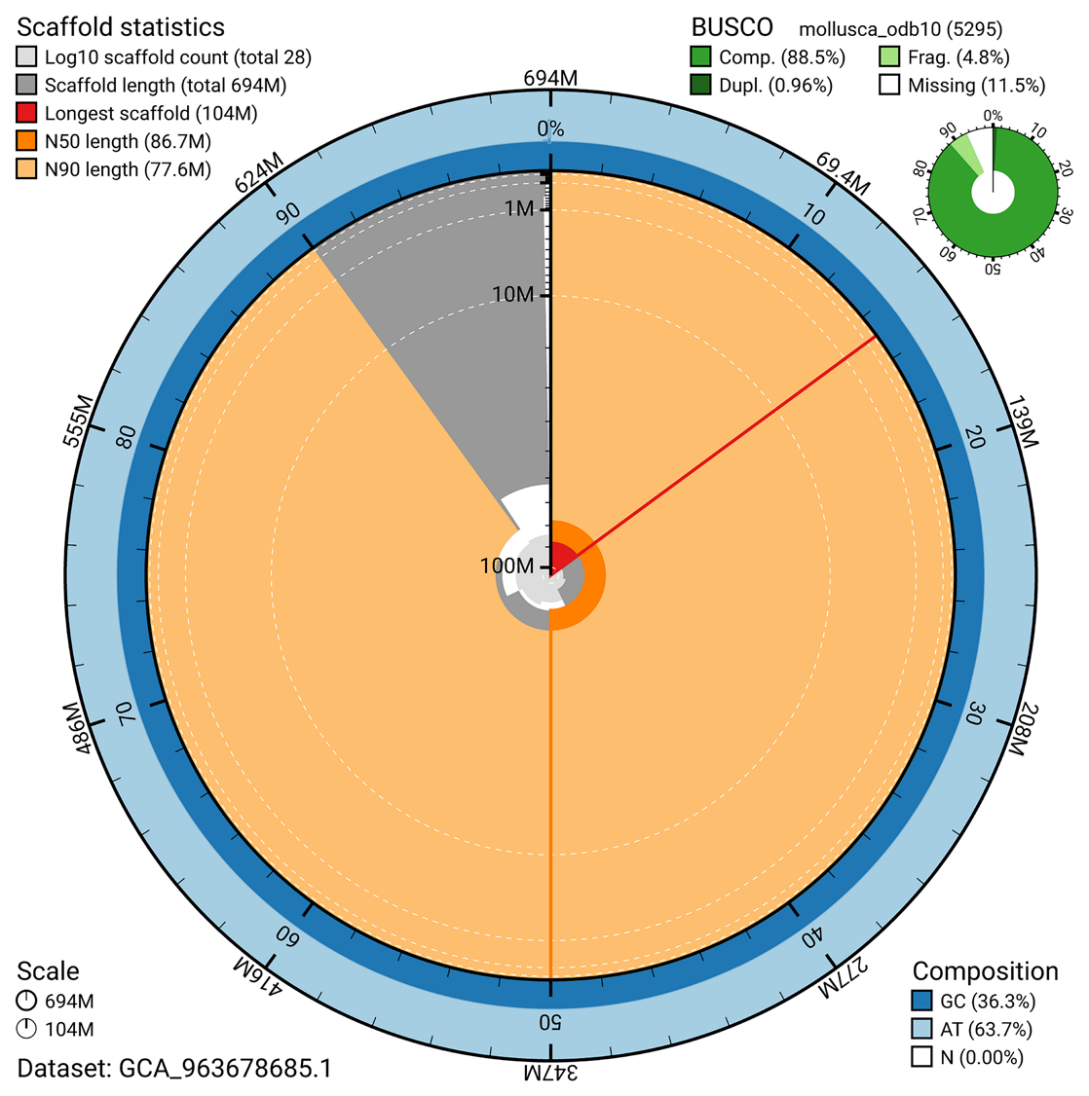
Assembly metrics for xgPatUlys2.1. The BlobToolKit snail plot provides an overview of assembly metrics and BUSCO gene completeness. The circumference represents the length of the whole genome sequence, and the main plot is divided into 1,000 bins around the circumference. The outermost blue tracks display the distribution of GC, AT, and N percentages across the bins. Scaffolds are arranged clockwise from longest to shortest and are depicted in dark grey. The longest scaffold is indicated by the red arc, and the deeper orange and pale orange arcs represent the N50 and N90 lengths. A light grey spiral at the centre shows the cumulative scaffold count on a logarithmic scale. A summary of complete, fragmented, duplicated, and missing BUSCO genes in the mollusca_odb10 set is presented at the top right. An interactive version of this figure can be accessed on the BlobToolKit viewer.

**Figure 6.**
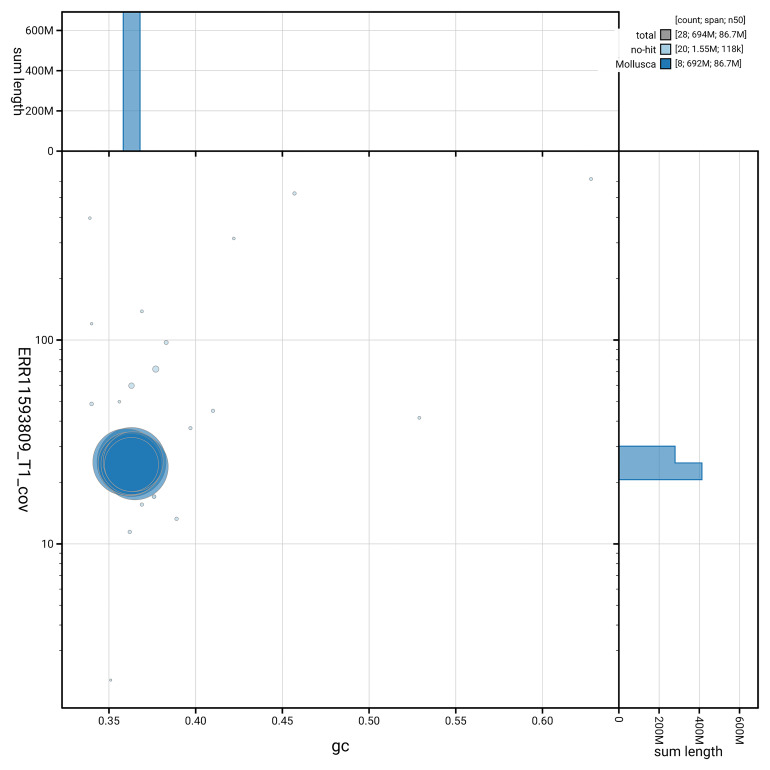
BlobToolKit GC-coverage plot for xgPatUlys2.1. Blob plot showing sequence coverage (vertical axis) and GC content (horizontal axis). The circles represent scaffolds, with the size proportional to scaffold length and the colour representing phylum membership. The histograms along the axes display the total length of sequences distributed across different levels of coverage and GC content. An interactive version of this figure is available on the BlobToolKit viewer.

The combined primary and alternate assemblies achieve an estimated QV of 64.2. The *k*-mer completeness is 72.96% for the primary assembly, 72.67% for the alternate haplotype, and 98.80% for the combined assemblies ( Figure 4). BUSCO analysis using the mollusca_odb10 reference set ( *n* = 5 295) identified 88.5% of the expected gene set (single = 87.5%, duplicated = 1.0%). The snail plot in Figure 5 summarises the scaffold length distribution and other assembly statistics for the primary assembly. The blob plot in Figure 6 shows the distribution of scaffolds by GC proportion and coverage.

Table 4 lists the assembly metric benchmarks adapted from Rhie *et al.* (2021) the Earth BioGenome Project Report on Assembly Standards September 2024. The EBP metric, calculated for the primary assembly, is **7.C.Q63**, meeting the recommended reference standard.

**Table 4.** Earth Biogenome Project summary metrics for the *Patella ulyssiponensis* assembly.

Measure	Value	Benchmark
EBP summary	7.C.Q63	6.C.Q40
Contig N50 length	16.75 Mb	≥ 1 Mb
Scaffold N50 length	86.69 Mb	= chromosome N50
Consensus quality (QV)	Primary: 63.6; alternate: 64.5; combined: 64.2	≥ 40
*k*-mer completeness	Primary: 72.96%; alternate: 72.67%; combined: 98.80%	≥ 95%
BUSCO	C:88.5%[S:87.5%; D:1.0%]; F:4.8%; M:6.7%; n:5 295	single > 90%; duplicated < 5%
Percentage of sequence assigned to chromosomes	99.78%	≥ 90%

## Genome annotation report

The *Patella ulyssiponensis* genome assembly (GCA_963678685.1) was annotated by Ensembl at the European Bioinformatics Institute (EBI). This annotation includes 61 607 transcribed mRNAs from 21 151 protein-coding and 22 298 non-coding genes. The average transcript length is 14 847.99 bp. There are 1.42 coding transcripts per gene and 5.85 exons per transcript. For further information about the annotation, please refer to the annotation page on Ensembl.

### Wellcome Sanger Institute – Legal and Governance

The materials that have contributed to this genome note have been supplied by a Darwin Tree of Life Partner. The submission of materials by a Darwin Tree of Life Partner is subject to the **‘Darwin Tree of Life Project Sampling Code of Practice’**, which can be found in full on the Darwin Tree of Life website. By agreeing with and signing up to the Sampling Code of Practice, the Darwin Tree of Life Partner agrees they will meet the legal and ethical requirements and standards set out within this document in respect of all samples acquired for, and supplied to, the Darwin Tree of Life Project.

Further, the Wellcome Sanger Institute employs a process whereby due diligence is carried out proportionate to the nature of the materials themselves, and the circumstances under which they have been/are to be collected and provided for use. The purpose of this is to address and mitigate any potential legal and/or ethical implications of receipt and use of the materials as part of the research project, and to ensure that in doing so we align with best practice wherever possible. The overarching areas of consideration are:

- Ethical review of provenance and sourcing of the material
- Legality of collection, transfer and use (national and international)

Each transfer of samples is further undertaken according to a Research Collaboration Agreement or Material Transfer Agreement entered into by the Darwin Tree of Life Partner, Genome Research Limited (operating as the Wellcome Sanger Institute), and in some circumstances, other Darwin Tree of Life collaborators.

## Data Availability

European Nucleotide Archive: Patella ulyssiponensis (China limpet). Accession number PRJEB63446. The genome sequence is released openly for reuse. The *Patella ulyssiponensis* genome sequencing initiative is part of the Darwin Tree of Life Project (PRJEB40665) and the Sanger Institute Tree of Life Programme (PRJEB43745). All raw sequence data and the assembly have been deposited in INSDC databases. Raw data and assembly accession identifiers are reported in Table 1 and Table 2. Pipelines used for genome assembly at the WSI Tree of Life are available at https://pipelines.tol.sanger.ac.uk/pipelines. Table 5 lists software versions used in this study.

## Author information

Members of the Marine Biological Association Genome Acquisition Lab are listed here: https://doi.org/10.5281/zenodo.15333539.

Members of the Wellcome Sanger Institute Tree of Life Management, Samples and Laboratory team are listed here: https://doi.org/10.5281/zenodo.12162482.

Members of Wellcome Sanger Institute Scientific Operations – Sequencing Operations are listed here: https://doi.org/10.5281/zenodo.14870789.

Members of the Wellcome Sanger Institute Tree of Life Core Informatics team are listed here: https://doi.org/10.5281/zenodo.12160324.

Members of the Tree of Life Core Informatics collective are listed here: https://doi.org/10.5281/zenodo.12205391.

Members of the Darwin Tree of Life Consortium are listed here: https://doi.org/10.5281/zenodo.4783558.
