# Supplementary material for: The genome sequence of the China Limpet, Patella ulyssiponensis Gmelin, 1791 (Patellidae)
Source: Wellcome Open Res. 2025 Aug 11;10:419. [Version 1] doi: 10.12688/wellcomeopenres.24681.1 (PMC12405849; doi:10.12688/wellcomeopenres.24681.1)
Supplement: Peer review history

# Reviewer report: Reviewer response for version 1

**DOI:** 10.21956/wellcomeopenres.27197.r130768

**Published:** 2025-09-17

**Authors:** Marco Gerdol

**Affiliations:** 1 University of Trieste, Trieste, Italy

This manuscript reports the high quality genome assembly of the limpet *Patella ulyssiponensis*. The work carried out by the authors follows gold standard methodologies and therefore there are no major methodological concerns about the assembly and annotation process. A couple of minor notes that may be added concern the exclusive use of muscle tissue for the generation of RNA-seq libraries, which might have prevented gathering sufficient evidence for the annotation of a number of genes expected to display high tissue specificity, or selectively expressed during embryonal stages. With this regard, BUSCO was only run in a genome mode and therefore it would be useful to also report the same statistics obtained while analysing the outcome of the annotation process in the “Genome annotation report” section.

 On a separate minor note, the introduction should clarify the apparently misleading common name of the species (i.e. China limpet), since the actual distribution range does not suggest an Asiatic origin.

Are sufficient details of methods and materials provided to allow replication by others?

Yes

Is the rationale for creating the dataset(s) clearly described?

Yes

Are the datasets clearly presented in a useable and accessible format?

Yes

Are the protocols appropriate and is the work technically sound?

Yes

Reviewer Expertise:

Molluscan genomics

I confirm that I have read this submission and believe that I have an appropriate level of expertise to confirm that it is of an acceptable scientific standard, however I have significant reservations, as outlined above.

# Reviewer report: Reviewer response for version 1

**DOI:** 10.21956/wellcomeopenres.27197.r130771

**Published:** 2025-09-02

**Authors:** José M Martín-Durán

**Affiliations:** 1 Queen Mary University of London, London, UK

This Genome Note reports the sequencing and assembly of the genome of Patella ulyssiponensis, a widely distributed limpet with important ecological implications. The methods and genome assembly are of high quality, making this an important resource for ecological and evolutionary studies. I only have a few minor points:

 - Patella ulyssiponensis in italics in Keywords.

 - Why is there a link in "remains uncertain" in the background?

Are sufficient details of methods and materials provided to allow replication by others?

Yes

Is the rationale for creating the dataset(s) clearly described?

Yes

Are the datasets clearly presented in a useable and accessible format?

Yes

Are the protocols appropriate and is the work technically sound?

Yes

Reviewer Expertise:

Comparative genomics, evolutionary biology, evo-devo

I confirm that I have read this submission and believe that I have an appropriate level of expertise to confirm that it is of an acceptable scientific standard.

# Reviewer report: Reviewer response for version 1

**DOI:** 10.21956/wellcomeopenres.27197.r130769

**Published:** 2025-09-02

**Authors:** Masa-Aki Yoshida

**Affiliations:** 1 Shimane University, Matsue, Shiman,, Japan

The full genome report has undergone thorough analysis and is considered satisfactory.

 The following are minor suggestions that should be addressed:

 ・The following statement likely requires a citation: In related species, sex change can depend on density; this has yet to be tested in P. ulyssiponensis.

 ・The BUSCO analysis results (in Assembly quality metrics) appear to fall below the EBP benchmark. It would be beneficial to include a statement indicating whether these values are comparable to typical figures for the closely related genus Patella.

Are sufficient details of methods and materials provided to allow replication by others?

Yes

Is the rationale for creating the dataset(s) clearly described?

Yes

Are the datasets clearly presented in a useable and accessible format?

Yes

Are the protocols appropriate and is the work technically sound?

Yes

Reviewer Expertise:

Evolutionary Biology, Genomics

I confirm that I have read this submission and believe that I have an appropriate level of expertise to confirm that it is of an acceptable scientific standard.

# Reviewer report: Reviewer response for version 1

**DOI:** 10.21956/wellcomeopenres.27197.r129506

**Published:** 2025-08-26

**Authors:** Ximing Guo

**Affiliations:** 1 Department of Marine and Coastal Sciences, Rutgers University New Brunswick, New Brunswick, New Jersey, USA

The article reports a genome for the china limpet *Patella ulyssiponensis.* The rationale for creating the database is clear and strong. Information on the distribution, ecology and biology of the species is helpful. *P. ulyssiponensis *is a member of a basal gastropod group that holds a unique position in gastropod evolution. A few sentences on its evolutionary significance would be helpful. 

 The approaches and protocols are appropriate and mostly sound. Some of the methods and results are not fully described. It is not clear how the genome is annotated and what the 22k non-coding genes are. Were the RNA-seq data used for gene annotation? Only one organ/tissue (muscle) was used for RNA-seq. How would the absence of other organs affect gene annotation? 

 The BUSCO score of the reported genome (88.5%) is low. Well-annotated molluscan genomes have BUSCO scores of 95-99%. Could the low BUSCO score be caused by inadequate gene annotation? 

 I did not find the annotated genome in gff3 or gff formats, and the protein and CDS sequence files. These data files are most useful.

Are sufficient details of methods and materials provided to allow replication by others?

Partly

Is the rationale for creating the dataset(s) clearly described?

Yes

Are the datasets clearly presented in a useable and accessible format?

No

Are the protocols appropriate and is the work technically sound?

Yes

Reviewer Expertise:

Molluscan genetics and genomics

I confirm that I have read this submission and believe that I have an appropriate level of expertise to confirm that it is of an acceptable scientific standard, however I have significant reservations, as outlined above.
